# Indoleamine 2,3-Dioxygenase-Dependent Neurotoxic Kynurenine Metabolism Contributes to Poststroke Depression Induced in Mice by Ischemic Stroke along with Spatial Restraint Stress

**DOI:** 10.1155/2018/2413841

**Published:** 2018-12-30

**Authors:** Young Soo Koo, Hyunha Kim, Jung Hwa Park, Min Jae Kim, Yong-Il Shin, Byung Tae Choi, Seo-Yeon Lee, Hwa Kyoung Shin

**Affiliations:** ^1^Department of Korean Medical Science, School of Korean Medicine, Pusan National University, Yangsan, Gyeongnam 50612, Republic of Korea; ^2^Korean Medical Science Research Center for Healthy-Aging, Pusan National University, Yangsan, Gyeongnam 50612, Republic of Korea; ^3^Graduate Training Program of Korean Medicine for Healthy-Aging, Pusan National University, Yangsan, Gyeongnam 50612, Republic of Korea; ^4^Department of Rehabilitation Medicine, School of Medicine, Pusan National University, Yangsan, Gyeongnam 50612, Republic of Korea; ^5^Research Institute for Convergence of Biomedical Science and Technology, Pusan National University Yangsan Hospital, Yangsan, Gyeongnam 50612, Republic of Korea

## Abstract

**Aim:**

Poststroke depression (PSD), which occurs in approximately one-third of stroke survivors, is clinically important because of its association with slow functional recovery and increased mortality. In addition, the underlying pathophysiological mechanisms are still poorly understood.

**Methods:**

We used a mouse model of PSD to examine the neurobiological mechanisms of PSD and the beneficial effects of aripiprazole, an atypical antipsychotic drug. PSD was induced in mice by combining middle cerebral artery occlusion (MCAO) with spatial restraint stress. The body weight, sucrose preference, and forced swim tests were performed at 5, 7, and 9 weeks and the Morris water maze test at 10 weeks after completing MCAO and spatial restraint stress.

**Results:**

Mice subjected to MCAO and spatial restraint stress showed significant depressive-like behavior in the sucrose preference test and forced swim test as well as cognitive impairment in the Morris water maze test. The PSD-like phenotype was accompanied by an indoleamine 2,3-dioxygenase 1 (IDO1) expression increase in the nucleus accumbens, hippocampus, and hypothalamus, but not in the striatum. Furthermore, the increased IDO1 levels were localized in Iba-1(+) cells but not in NeuN(+) or GFAP(+) cells, indicating that microglia-induced IDO1 expression was prominent in the PSD mouse brain. Moreover, 3-hydroxyanthranilate 3,4-dioxygenase (HAAO), quinolinic acid (QUIN), and reactive oxygen species (ROS) were significantly increased in the nucleus accumbens, hippocampus, and hypothalamus of PSD mice. Importantly, a 2-week aripiprazole (1 mg/kg, *per os*) regimen, which was initiated 1 day after MCAO, ameliorated depressive-like behavior and impairment of cognitive functions in PSD mice that was accompanied by downregulation of IDO1, HAAO, QUIN, and ROS.

**Conclusions:**

Our results suggest that the IDO1-dependent neurotoxic kynurenine metabolism induced by microglia functions in PSD pathogenesis. The beneficial effect of aripiprazole on depressive-like behavior and cognitive impairment may be mediated by inhibition of IDO1, HAAO, QUIN, and ROS.

## 1. Introduction

Poststroke depression (PSD) is a prevalent condition, affecting about 33% of stroke survivors [1]. Although the development of PSD varies depending on the type and the time since stroke, the incidence rate of nearly 1 in 3 stroke survivors is highest during the first year but declines thereafter [2]. PSD is characterized by increased cognitive deficits, social withdrawal, insomnia, anhedonia, and despair feelings [3]. In addition, it is associated with poor functional recovery and quality of life [4] and increased risk of recurrent stroke and death [5]. Although a high proportion of stroke patients progress to PSD, the underlying neurobiological mechanisms have not yet been thoroughly investigated.

A reliable chronic animal model of depression after stroke has to be carefully selected for studies on the mechanisms underlying PSD. Currently, the most commonly used type of PSD models is the combination of experimental ischemic lesions and social isolation or unpredictable chronic mild stress [6, 7]. Moreover, a single middle cerebral artery (MCA) occlusion (MCAO) procedure also leads to anhedonia, despair, or cognitive impairment, suggesting that ischemic lesions may directly affect neural circuits involved in mood regulation and contribute to the susceptibility to PSD [8]. However, we do not know if this type of model truly reflects the progression of the clinical events in PSD. A recently proposed PSD animal model combines MCAO with spatial restraint stress [9]. Stroke patients can experience secondary physical and psychological stress because they developed a movement disorder as a documented complication of stroke. Therefore, the combination of MCAO and spatial restraint stress represents an ideal model for studying the mechanisms of PSD and experimental therapies of PSD because it includes a restrictive movement parameter.

Regarding the etiology of PSD, there appears to be a multifactorial rather than a simple biological or psychological cause. A meta-analysis study identified cerebral perfusion reduction, higher cortisol levels, low levels of neurotrophic factors, and amygdala volume reduction as potential biological markers in PSD patients [10]. In addition, animal studies have demonstrated that several biological factors may contribute to the development of PSD, such as neuroinflammation, alterations in neurotrophic factors, disruption of neural networks, and neuroendocrine dysregulation triggered by brain ischemia [8, 11, 12]. However, the specific pathophysiology of PSD is still open to debate and an effective pharmacotherapy has not yet been developed for PSD.

Several studies have indicated a role of proinflammatory cytokines in the development of PSD [13, 14]. Furthermore, increased levels of inflammatory cytokines reduce the synthesis and availability of serotonin via their enhancing effect on the activity of indoleamine 2,3-dioxygenase 1 (IDO1) [14]. Many studies have demonstrated that the upregulation of IDO1 by proinflammatory cytokines induces depressive-like behaviors [15, 16]. A recent study identified the IDO1-dependent neurotoxic kynurenine metabolism as a pathogenic factor for cognitive dysfunction in inflammation-induced depressive disorders and a potential novel target for the treatment of these disorders [17]. However, few studies have examined whether the IDO1-dependent neurotoxic kynurenine metabolism is involved in PSD progression.

In the current study, we examined the roles of IDO1 and IDO1-dependent neurotoxic kynurenine metabolism using the combination of MCAO along with spatial restraint stress to induce PSD in mice. We hypothesized that IDO1-dependent neurotoxic kynurenine metabolite, quinolinic acid (QUIN), and reactive oxygen species (ROS) would lead to PSD-like behavior and examined the involvement of IDO1, QUIN, and ROS in mediating this behavioral effect. Then, we investigated the effect of aripiprazole on the behavioral changes and the production of IDO1, QUIN, and ROS. These studies will improve our understanding of the pathophysiological mechanism underlying PSD.

## 2. Materials and Methods

### 2.1. Animals

Male C57BL/6 mice, 6 weeks of age, were purchased from DooYeol Biotech (Seoul, Korea). The mice were housed in a facility with a 12 h light-dark cycle at 22°C and given *ad libitum* access to food and tap water. All animal experiments were conducted in accordance with the ethical and scientific procedures described in guidelines of the Pusan National University-Institutional Animal Care and Use Committee (PNU-IACUC) and approved by the PNU-IACUC at the Pusan National University (approval number PNU-2016-1182 and PNU-2017-1557).

### 2.2. Focal Cerebral Ischemia

Focal cerebral ischemia was induced by MCAO using a previously described intraluminal filament technique [18]. Anesthesia was achieved using nose cone-delivered isoflurane (maintained at 1.5% in 80% N_2_O and 20% O_2_). Regional cerebral blood flow (CBF) was measured by a fiber-optic probe affix to the skull over the MCA using a PeriFlux Laser Doppler System 5000 (Perimed, Stockholm, Sweden). MCAO was induced by a silicon rubber-coated 7-0 monofilament (Doccol Corporation, Redlands, CA) in the internal carotid artery, after which the monofilament was advanced to occlude the MCA. In all animals, the regional CBF was measured to confirm consistent and similar levels of ischemic induction. The filament was withdrawn 45 min after occlusion, and reperfusion was confirmed by laser Doppler monitoring. The surgical wound was sutured, and mice were allowed to recover from anesthesia. Rectal temperature was maintained at 36.5–37.5°C using a Panlab thermostatically controlled heating mat (Harvard Apparatus, Holliston, MA, USA) throughout the procedure from the start of the surgery until the animals recovered from anesthesia.

### 2.3. Spatial Restraint Stress

**Figure 1:**
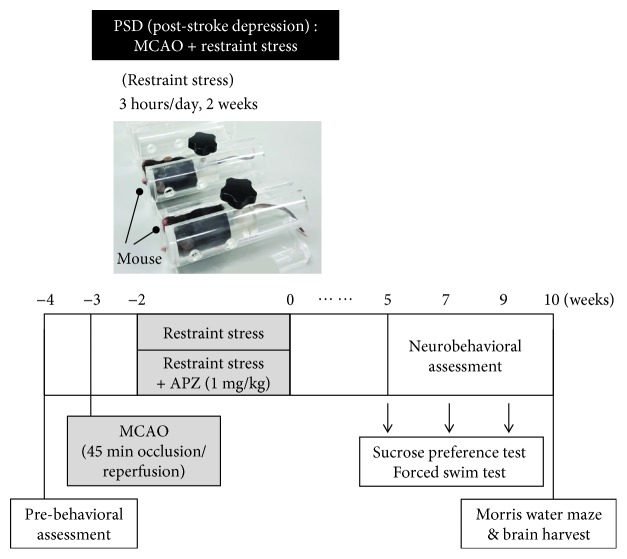
Timeline of events in animal experiment. A mouse model of poststroke depression (PSD) was induced by combining the middle cerebral artery occlusion (MCAO) with spatial restraint stress. After a recovery period of 1 week after MCAO surgery, spatial restraint stress was performed on 14 consecutive days. During the 2 weeks of spatial restraint stress, the PSD mice were treated with either aripiprazole (1 mg/kg) or vehicle orally once per day. The body weight (appetite), sucrose preference test (anhedonia), and forced swim test (despair-like behavior) were conducted at 5, 7, and 9 weeks and the Morris water maze test at 10 weeks after completing MCAO and spatial restraint stress.

Spatial restraint stress exposure was initiated on the 7th day after MCAO surgery and performed on 14 consecutive days (Figure 1). Mice were individually placed into well-ventilated custom-made tubes every day for 3 h (from 9 : 30 am to 12 : 30 pm) without being able to move forward or backward. After completion of the spatial restraint stress exposure, the mice were removed from the tubes and returned to their cages. The timeline of events is shown in Figure 1.

### 2.4. Drug Administration

Aripiprazole was donated by Otsuka Pharmaceutical (Tokushima, Japan). Starting 1 week after MCAO, during the 2-week spatial restraint stress exposure, PSD mice assigned for drug treatment received a daily dose of aripiprazole by oral gavage (Figure 1). Aripiprazole was administered at a dose of 1 mg/kg in 20% DMSO (Duchefa Biochemie, Haarlem, Netherlands). PSD mice assigned to the vehicle group received a daily sham treatment of 20% DMSO according to the same treatment regimen used for the aripiprazole-treated mice.

### 2.5. Behavior Tests

The body weight, sucrose preference, and forced swim tests were performed at weeks 5, 7, and 9 and the Morris water maze test at week 10 after completing MCAO and spatial restraint stress.

#### 2.5.1. Sucrose Preference Test

The sucrose preference test was performed to measure anhedonia caused by PSD in mice. The mice were given access to both water and a sucrose solution, and their preference for the sucrose solution was quantified. Briefly, mice were deprived of food and water for 20 h. One bottle of water and one containing 1% sucrose were simultaneously placed in the cages and were freely accessible to the mice for 3 h. The position of the two bottles (left or right side of the cage) was varied randomly from trial to trial. The volume of each liquid was measured before and after each trial, and sucrose preference was calculated according to the following equation: sucrose preference = (sucrose consumption)/(sucrose consumption + water consumption) × 100.

#### 2.5.2. Forced Swim Test

The forced swim test was performed to measure despair-like behavior [19]. One day before the first test, mice were exposed to 23–25°C water for 1 min in a glass cylinder (20 cm in height × 15 cm diameter). The forced swim test was recorded using a digital camera (E8400, Nikon Corporation, Tokyo, Japan) for 6 minutes. After the initial 2 min of vigorous activity, the behavior (immobility by floating in the water without struggling and doing only those movements necessary to keep the head above the water) was scored during the last 4 min.

#### 2.5.3. Morris Water Maze Test

The Morris water maze test was conducted to evaluate the effect of memory dysfunction [20]. A circular target platform (10 × 10 cm) was immersed in a pool (diameter 120 cm, depth 50 cm), and a high-contrast cue was attached to the inside of the pool near the platform above the water surface. The water temperature was maintained at 20–21°C. The test was conducted every day for 7 consecutive days. On day 1, before starting the main experiment, all mice were free to swim with the cue and visible platform in a trial for 90 seconds to adapt to the water. On days 2–6, each mouse was trained five times per day for 5 consecutive days in hidden platform trials using opaque water. When the mouse found the platform within 90 s, the mouse was allowed to view the cues on the platform for 15 s. If the platform was not found by the mouse within 90 s, the mouse was guided to the platform and allowed to view the cues on the platform for 30 s. On day 7, the platform was removed from the pool, and the probe trial test was performed for 90 s. The swimming was video-tracked. Travel distance and latency were measured in the quadrant where the platform was located using Smart software (Panlab, Barcelona, Spain).

### 2.6. Immunofluorescence Staining

Mice were deeply anesthetized with sodium thiopental and subsequently perfused transcardially with cold phosphate-buffered saline (PBS), followed by 4% paraformaldehyde for fixation. Each mouse brain was removed and further fixed in 4% paraformaldehyde at 4°C for 24 h, followed by cryoprotection in 30% sucrose for 72 h at 4°C. Next, the isolated brains were frozen and stored at −80°C until examination. The frozen brains were sliced at a thickness of 40 *μ*m using a CM3050 cryostat (Leica Microsystems, Wetzlar, Germany) and stored in a storage solution (50% glycerol in PBS, pH 7.4) at −20°C. The brain sections were incubated with the following primary antibodies, rat anti-IDO1 (1 : 200, sc-53978, Santa Cruz Biotechnology, Santa Cruz, CA), rabbit anti-3-hydroxyanthranilate 3,4-dioxygenase (HAAO) (1 : 200, ab106436, Abcam, Cambridge, UK), rabbit anti-QUIN (1 : 100, ab37106, Abcam), mouse anti-GFAP (1 : 200, Z0334, Dako, Glostrup, Denmark), rabbit anti-NeuN (1 : 200, ab133303, Abcam), rabbit anti-Iba-1 (1 : 200, 019-19741, Wako, Osaka, Japan), and rabbit anti-BDNF (1 : 200, SC-546, Santa Cruz Biotechnology) at 4°C overnight. The samples were incubated for immunostaining with Alexa 488 (Invitrogen, Carlsbad, CA, USA) or Alexa 594-conjugated secondary antibodies (Invitrogen) for 2 hours in the dark. Nuclei were counterstained with DAPI (Molecular Probes, Eugene, OR, USA). Section images were captured with a laser scanning microscope (LSM 700, Carl Zeiss, Oberkochen, Germany). Morphological analysis and quantification of positive cells were conducted in a blinded manner using the iSolution analysis software (Image & Microscope Technology, Vancouver, Canada). For quantification of positive cells, at least three randomly selected fields per three adjacent brain sections from each mouse were examined and averaged.

### 2.7. Detection of Superoxide Anion

Reactive oxygen species production in the brain was assessed using *in vivo* dihydroethidium (DHE, Life Technologies, Eugene, OR) staining. DHE, a cell-permeable oxidation-sensitive fluorescent dye, is oxidized to ethidium by superoxide, which, subsequently, binds to DNA in the nucleus and emits red fluorescence. The frozen brain samples were sliced at a thickness of 40 *μ*m using a CM3050 cryostat (Leica Microsystems) and incubated with DHE (50 *μ*M) in PBS for 10 min at 37°C in a humidified chamber protected from light. The images of each section were captured with a laser scanning microscope (LSM 700, Carl Zeiss), and quantification of DHE-positive cells in three coronal sections of each animal was performed using the iSolution analysis software (Image & Microscope Technology).

### 2.8. Corticosterone Measurement

The corticosterone levels in the serum were analyzed using a commercial Corticosterone ELISA kit according to the manufacturer's instruction (Enzo Life Sciences, Bloomberg, Switzerland).

### 2.9. Statistical Analysis

Data are expressed as mean ± SEM. The differences between control and PSD groups were evaluated using an unpaired *t*-test. One-way ANOVA or two-way ANOVA with Tukey's post hoc comparison was used for statistical analysis comparing more than two groups. Statistical analyses were performed using SigmaPlot statistical program version 11.2 (Systat Software, San Jose, CA, USA). *P* < 0.05 was considered statistically significant.

## 3. Results

### 3.1. Analysis of PSD-Associated Behavior

**Figure 2:**
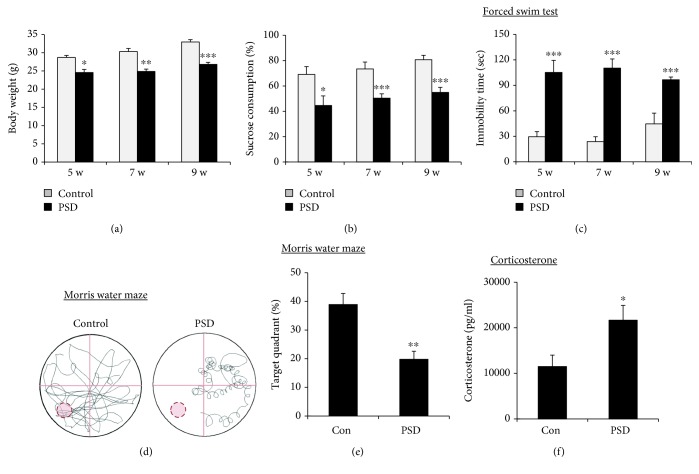
Spatial restraint stress after MCAO showed depressive-like behaviors and cognitive impairment. Body weight (a), sucrose preference test (b), and forced swim test (c) were performed at 5, 7, and 9 weeks after completing MCAO and spatial restraint stress. In the Morris water maze test, representative swimming traces (d) and percent of time spent (e) in the target quadrant where the hidden platform was previously placed during the probe trial session at 10 weeks after MCAO. (f) Corticosterone levels in serum were determined by commercial ELISA. Data are expressed as mean ± SEM (*N* = 5–6). ^∗^ *P* < 0.05, ^∗∗^ *P* < 0.01, and ^∗∗∗^ *P* < 0.001 vs. control.

To validate the induction of PSD in mice, we assessed the body weight, sucrose preference test, and forced swim test at 5, 7, and 9 weeks and the Morris water maze test at 10 weeks after completion of MCAO and spatial restraint stress (Figure 1). Body weight gain is an indicator of appetite that was markedly decreased in the PSD mice (Figure 2(a)). As an indication of anhedonic behavior, the consumption of a 1% sucrose solution was significantly decreased in PSD mice as compared to that in control mice (Figure 2(b)). Moreover, the immobility time in the forced swim test, which is a measure of despair-like behavior, was significantly longer in the PSD mice than that in the control mice (Figure 2(c)). In the Morris water maze test, the PSD mice spent less time in the target quadrant than the control group, suggesting that the PSD group had a significant impairment in spatial learning and memory (Figures 2(d) and 2(e)). Because a meta-analysis suggested that increased cortisol levels and reduced levels of neurotrophic factors may represent potential biomarkers for PSD [10], we measured the levels of corticosterone in serum and brain-derived neurotrophic factor (BDNF) in the brain. The corticosterone level in serum was significantly increased (Figure 2(e)) and the BDNF expression in the brain was markedly reduced (Supplementary Figure S1) in the PSD group as compared to those in the control group. These results suggested that the MCAO mice exposed to restraint stress developed severe changes in appetite, anhedonia, and despair-like behavior with cognitive impairment and exhibited biological markers for PSD.

### 3.2. IDO1 and HAAO Expression and QUIN Production after PSD

**Figure 3:**
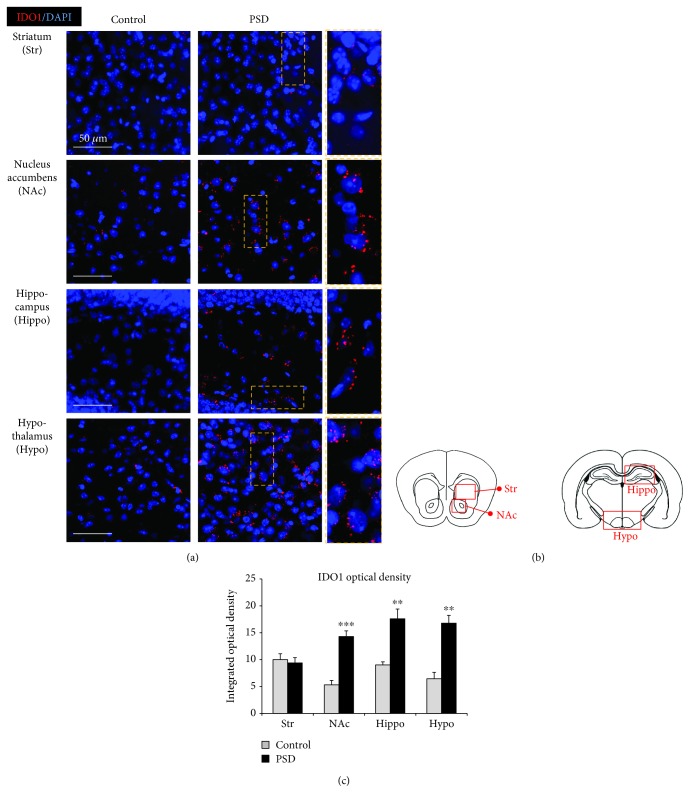
IDO1 expression in the brain of PSD mice. (a) Representative images of immunofluorescent staining for indoleamine 2,3-dioxygenase 1 (IDO1) in the striatum (Str), nucleus accumbens (NAc), hippocampus (Hippo), and hypothalamus (Hypo). Significantly higher levels of IDO1 immunoreactivity were observed in NAc, Hippo, and Hypo, but not in the Str of PSD mice. IDO1 was labeled with red fluorescence, and nuclear DNA was labeled with blue fluorescence by DAPI. The yellow dashed rectangle marks the position of an enlarged image of IDO1. Scale bar = 50 *μ*m. (b) Schematic diagram shows the regions of the striatum (Str), nucleus accumbens (NAc), hippocampus (Hippo), and hypothalamus (Hypo) of the brain. (c) Quantification of IDO1 fluorescence intensity. Data are expressed as mean ± SEM (*N* = 4). ^∗∗^ *P* < 0.01 and ^∗∗∗^ *P* < 0.001 vs. control.

**Figure 4:**
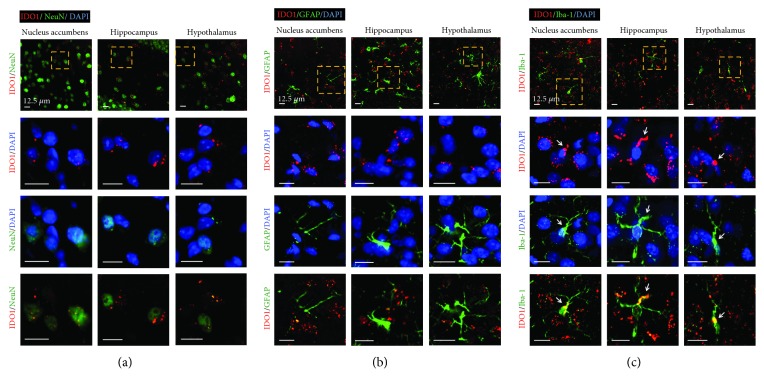
Double immunostaining for IDO1 and NeuN, GFAP, or Iba-1 in PSD mice. (a) Immunofluorescence of IDO1 (red) and NeuN (green), a neuronal marker of the nucleus accumbens, hippocampus, and hypothalamus in PSD mice. The yellow dashed rectangle marks the position of an enlarged image showing double immunostaining of IDO1 and NeuN. (b) Immunofluorescence of IDO1 (red) and GFAP (green), an astrocyte marker in PSD mice. The yellow dashed rectangle marks the position of an enlarged image showing double immunostaining of IDO1 and GFAP. (c) Immunofluorescence of IDO1 (red) and Iba-1 (green), a microglial marker in PSD mice. The yellow dashed rectangle marks the position of an enlarged image showing double immunostaining of IDO1 and Iba-1. Arrows indicate the colocalization of IDO-1(+)/Iba-1(+) immunofluorescence (yellow). Nuclear DNA was labeled with blue fluorescence by DAPI. Scale bar = 12.5 *μ*m.

**Figure 5:**
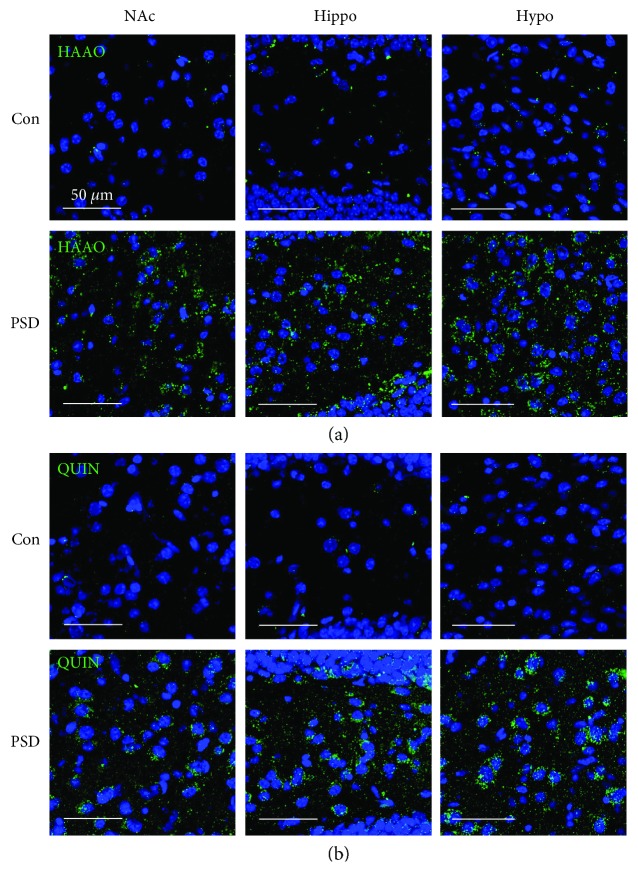
HAAO expression and QUIN production in the brain of PSD mice. Representative images of immunofluorescent staining of 3-hydroxyanthranilate 3,4-dioxygenase (HAAO) (a) and quinolinic acid (QUIN) (b) in the nucleus accumbens (NAc), hippocampus (Hippo), and hypothalamus (Hypo) of control and PSD mice. Higher levels of HAAO and QUIN were observed in PSD mice as compared with those in control mice. HAAO and QUIN were labeled with green fluorescence, and nuclear DNA was labeled with blue fluorescence by DAPI. Scale bar = 50 *μ*m.

Next, we examined IDO1 expression in different brain regions linked to PSD behaviors including striatum, nucleus accumbens, hippocampus, and hypothalamus. Significantly higher levels of IDO1 immunoreactivity were observed in the nucleus accumbens (269 ± 20%), hippocampus (195 ± 20%), and hypothalamus (260 ± 23%), but not in the striatum (94 ± 10%) of the PSD mice as compared to those in the respective brain regions of the control mice (Figure 3). Then, we investigated whether IDO1 immunoreactivity in the brain of PSD group mice was colocalized with NeuN (neuronal marker), GFAP (astrocyte marker), or Iba-1 (microglial marker) (Table 1). We found that IDO1 was primarily expressed in microglial cells (Figure 4(c)) and to a lesser extent in neuronal cells (Figure 4(a)) or astrocytes (Figure 4(b)). These results indicated that microglia-induced IDO1 production might be involved in PSD pathogenesis. Next, HAAO and QUIN, a major neurotoxic metabolite of the IDO1-dependent pathway in microglia, were detected by immunofluorescence staining. Higher levels of HAAO and QUIN were observed in the nucleus accumbens, hippocampus, and hypothalamus of PSD mice than those of control mice (Figure 5). These results suggested that IDO1-dependent neurotoxic kynurenine metabolite production may be linked to the development of PSD.

### 3.3. Behavioral Analysis for PSD after Aripiprazole Treatment

**Figure 6:**
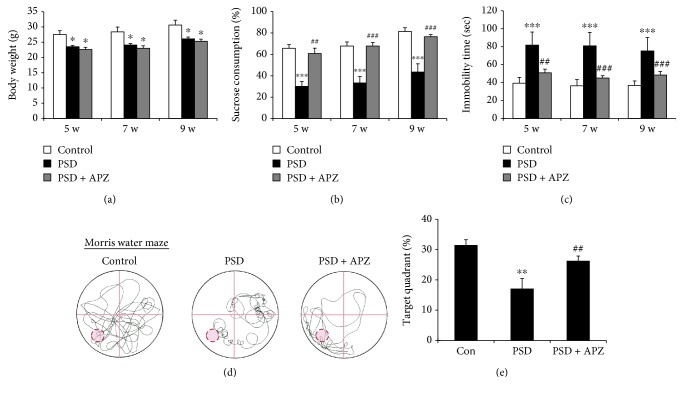
Effect of aripiprazole on depressive-like behaviors and cognitive impairment in PSD mice. During 2 weeks of spatial restraint stress, the PSD mice were treated with either aripiprazole (1 mg/kg) or vehicle orally once per day. The body weight (a), sucrose preference test (b), and forced swim test (c) were performed at 5, 7, and 9 weeks after completing MCAO and spatial restraint stress; control, sham (PSD), and aripiprazole-treated group (PSD + APZ). In the Morris water maze test, representative swimming traces (d) and percent of time spent (e) in the target quadrant where the hidden platform was previously placed during the probe trial session at 10 weeks after completing MCAO and spatial restraint stress. Depressive behaviors and cognitive impairment in the PSD model were restored by aripiprazole treatment. Data are expressed as mean ± SEM (*N* = 6–11). ^∗^ *P* < 0.05, ^∗∗^ *P* < 0.01, and ^∗∗∗^ *P* < 0.001 vs. control; ^##^ *P* < 0.01 and ^###^ *P* < 0.001 vs. PSD.

We examined whether the depressive behavior and cognitive impairment of the PSD mice could be improved by the antidepressant drug aripiprazole. During 2 weeks of spatial restraint stress, the mice in the PSD group were orally treated once per day with either aripiprazole (1 mg/kg) or vehicle (Figure 1). The body weight of aripiprazole-treated mice did not differ from that of sham mice (Figure 6(a)). However, both the sucrose preference and the forced swim test were significantly attenuated in the aripiprazole-treated group as compared with that in the vehicle group (Figures 6(b) and 6(c)). In addition, cognitive dysfunction caused by PSD was restored by aripiprazole treatment (Figures 6(d) and 6(e)), and the reduced expression of BDNF in the nucleus accumbens of PSD mice was reversed by aripiprazole (Supplementary Figure S1). In contrast, corticosterone levels did not differ between the aripiprazole-treated group and the vehicle group (data not shown). These results indicated that in mice with PSD, which was induced by restraint stress after MCAO, the depressive behavior and cognitive impairment were improved by aripiprazole treatment.

### 3.4. Effect of Aripiprazole Treatment on IDO1 and HAAO Expression and QUIN Production in PSD Mice

**Figure 7:**
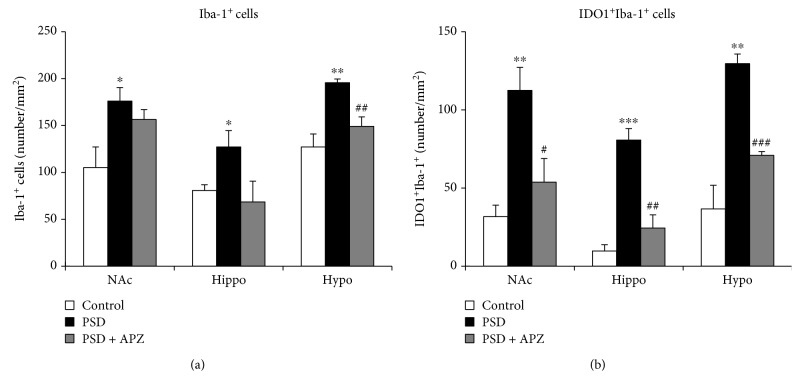
Effect of aripiprazole on Iba-1(+) cells or IDO1(+)/Iba-1(+) cells in PSD mice. The number of Iba-1(+) cells (a) or IDO(+)/Iba-1(+) cells (b) was determined by immunofluorescent staining of the nucleus accumbens (NAc), hippocampus (Hippo), and hypothalamus (Hypo) from control, sham (PSD), and aripiprazole-treated mice (PSD + APZ). Aripiprazole-treated PSD mice showed markedly lower numbers of IDO(+)/Iba-1(+) cells than did PSD mice. Data are expressed as mean ± SEM (*N* = 4). ^∗^ *P* < 0.05, ^∗∗^ *P* < 0.01, and ^∗∗∗^ *P* < 0.001 vs. control; ^#^ *P* < 0.05, ^##^ *P* < 0.01, and ^###^ *P* < 0.001 vs. PSD.

**Figure 8:**
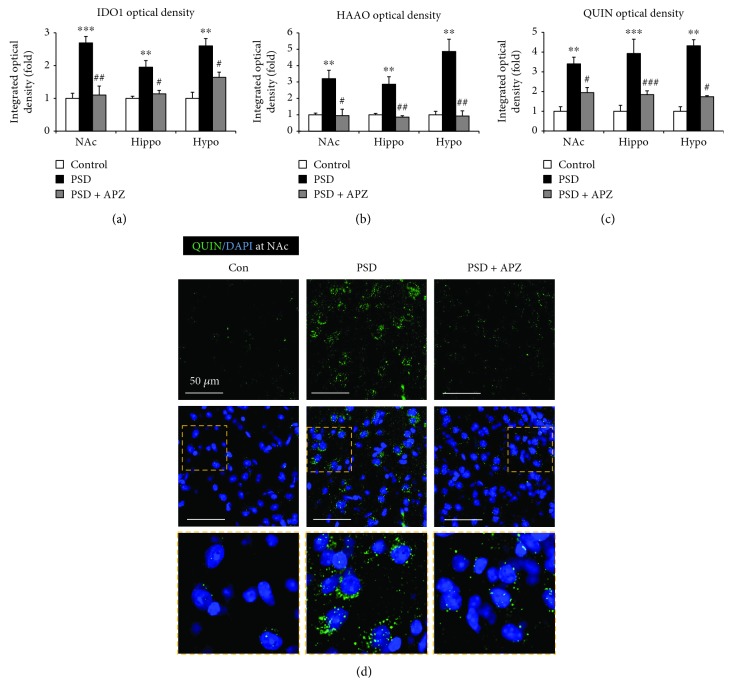
Effect of aripiprazole on IDO1 and HAAO expressions and QUIN production in the brain of PSD mice. IDO1 (a), HAAO (b), and QUIN (c) fluorescence intensities were determined by immunofluorescent staining of the nucleus accumbens (NAc), hippocampus (Hippo), and hypothalamus (Hypo) from control, sham (PSD), and aripiprazole-treated mice (PSD + APZ). Increased IDO1 and HAAO expression and QUIN production by PSD were reversed by aripiprazole treatment. Data are expressed as mean ± SEM (*N* = 4). ^∗∗^ *P* < 0.01 and ^∗∗∗^ *P* < 0.001 vs. control; ^#^ *P* < 0.05, ^##^ *P* < 0.01 and ^###^ *P* < 0.001 vs. PSD. (d) Representative images of immunofluorescent staining of QUIN in the nucleus accumbens (NAc) of control (Con), sham (PSD), and aripiprazole-treated mice (PSD + APZ). The yellow dashed rectangle marks the position of an enlarged image of QUIN. Nuclear DNA was labeled with blue fluorescence by DAPI. Scale bar = 50 *μ*m.

Our results suggested that microglia-induced IDO1 production might be involved in PSD pathogenesis. To determine whether the behavioral improvement in aripiprazole-treated PSD mice was due to the inhibition of microglial activation or microglia-induced IDO1 expression, we examined the effect of aripiprazole on Iba-1(+) cells or IDO1(+)/Iba-1(+) cells by immunofluorescence staining. The Iba-1(+) cells and IDO1(+)/Iba-1(+) cells in the nucleus accumbens, hippocampus, and hypothalamus were significantly increased in the PSD group as compared with those in the control group (Figures 7(a) and 7(b)). The aripiprazole-treated group showed a significantly lower number of Iba-1(+) cells in the hypothalamus than that of the vehicle group, while aripiprazole had no effect on Iba-1(+) cells in the nucleus accumbens. In addition, there was a trend for a lower number of Iba-1(+) cells in the hippocampus of aripiprazole-treated mice than that of vehicle-treated mice, although this difference was not statistically significant (Figure 7(a)). However, the number of IDO1(+)/Iba-1(+) cells was markedly reduced in aripiprazole-treated PSD mice than those in sham mice (Figure 7(b)), suggesting that aripiprazole mainly inhibited the microglia-induced IDO1 expression. We also investigated the effect of aripiprazole on IDO1 and HAAO expression and QUIN production using immunofluorescent analysis. PSD-induced increases in IDO1 and HAAO expression and QUIN production of PSD mice were significantly reversed by aripiprazole treatment as compared with those of sham mice (Figure 8). In combination with the behavioral data, our results indicated that improvement of PSD behavior by aripiprazole treatment might be mediated by the regulation of IDO1 and IDO1-dependent kynurenine metabolite production.

### 3.5. Effect of Aripiprazole Treatment on ROS Production in PSD Mice

**Figure 9:**
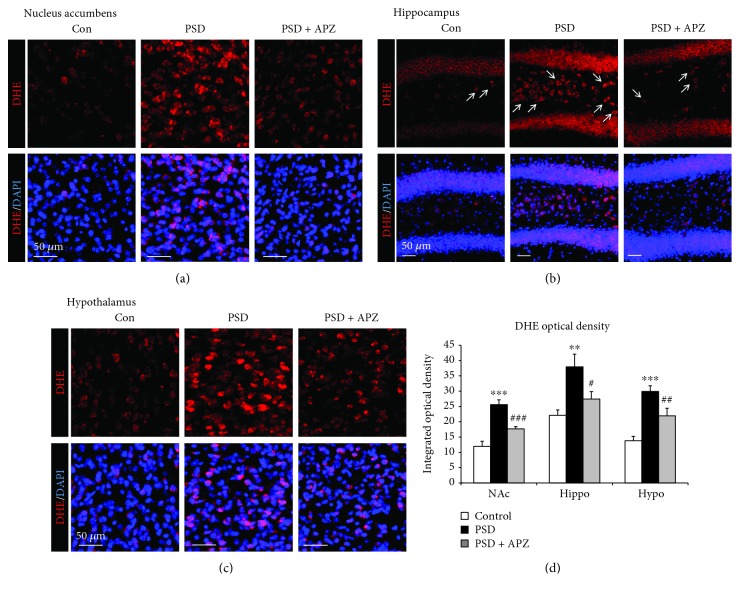
Effect of aripiprazole on ROS production in the brain of PSD mice. Reactive oxygen species (ROS) production was assessed by dihydroethidium (DHE) fluorescence staining. Representative photomicrographs of the fluorescence of DHE oxidation in the nucleus accumbens (a), hippocampus (b), and hypothalamus (c) of control (Con), sham (PSD), and aripiprazole-treated mice (PSD + APZ). The fluorescence of DHE oxidation is shown as red fluorescence (arrows), and nuclear DNA was labeled with blue fluorescence by DAPI. Scale bar = 50 *μ*m. (d) Quantification graphs of DHE fluorescence intensity. Data are expressed as mean ± SEM (*N* = 4). ^∗∗^ *P* < 0.01 and ^∗∗∗^ *P* < 0.001 vs. control; ^#^ *P* < 0.05, ^##^ *P* < 0.01, and ^###^ *P* < 0.001 vs. PSD.

Because quinolinic acid may be neurotoxic due to increased oxidative stress [21, 22], we examined ROS production using DHE, a marker for superoxide (Figure 9). The intensity of red fluorescence of DHE-positive cells was markedly increased in the nucleus accumbens, hippocampus, and hypothalamus of PSD group mice relative to those of control group mice. The mice of the aripiprazole-treated group showed a red fluorescence intensity that was significantly lower than that of the sham group, indicating that aripiprazole treatment attenuated the oxidative stress increased by PSD.

## 4. Discussion

**Figure 10:**
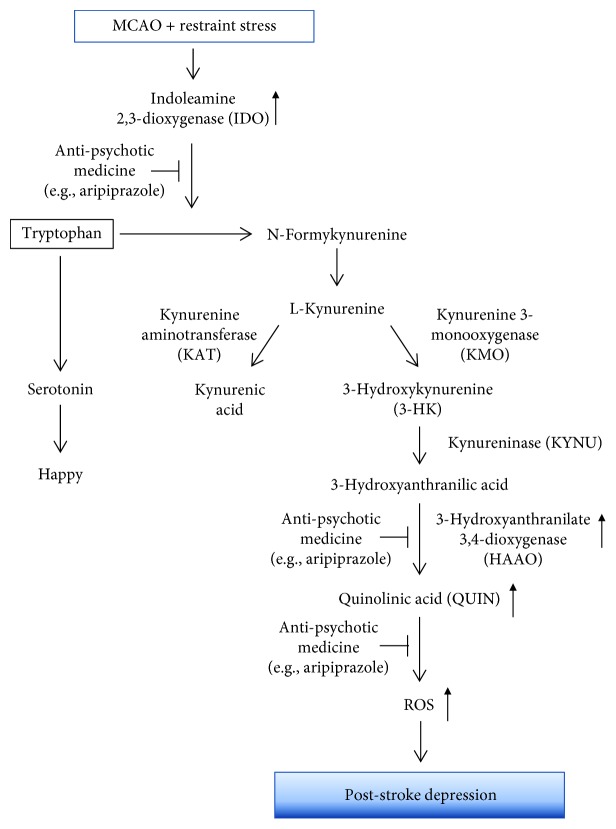
Schematic illustration of the potential mechanism by which IDO1 and IDO1-dependent kynurenine metabolite, QUIN, are involved in PSD animal models and the therapeutic effects of antidepressant aripiprazole.

The present study was designed to evaluate the role of IDO1, IDO1-dependent neurotoxic kynurenine metabolite QUIN, and ROS as pathogenic mediators in a mouse model of PSD. The mice exposed to MCAO and spatial restraint stress exhibited depressive-like behavior, while microglial IDO1 expression, QUIN production, and ROS were prominent in the nucleus accumbens, hippocampus, and hypothalamus of these mice. The adjunctive antidepressant aripiprazole ameliorated depressive behavior and cognitive impairment in the PSD mice via downregulation of IDO1, HAAO, QUIN, and ROS. Our study provides new insight into the summative pathogenesis of spatial restraint stress after MCAO, suggesting that the IDO1-dependent neurotoxic kynurenine metabolism may represent a potential therapeutic target for the treatment of PSD (Figure 10).

The sucrose preference and forced swim tests are widely accepted behavioral parameters for assessing depression and antidepressant-like effects in rodents [6]. Reduced sucrose intake in rodents is frequently used as an index of anhedonia whereas the forced swim test measures the immobility of depressed animals in a despair situation. In our study, we noticed significant reductions in body weight gain and sucrose intake and an increase in immobility in the PSD mice as compared to those in the control mice at 5, 7, and 9 weeks after completion of MCAO and spatial restraint stress exposure (Figures 2(a)–2(c)).

Interestingly, no significant depressive-like behavior was observed prior to 5 weeks after completing MCAO and spatial restraint stress, indicating that the depressive-like behavior was the delayed effect of MCAO and spatial restraint stress. However, these results were not consistent with the previous results that reported depressive-like behavior beginning at 2 weeks after MCAO and spatial restraint stress [9]. This discrepancy was probably due to some experimental differences such as the ischemia time (60 min MCAO in the previous study vs. 45 min MCAO in this study), the initiation time point of spatial restraint stress (3 days after MCAO vs. 7 days after MCAO) and its duration (2 hours/day vs. 3 hours/day), and the mouse species (ICR vs. C57BL/6 mice). Moreover, in the Morris water maze test, the mice exposed to MCAO and spatial restraint stress spent a lower percentage of time in the target quadrant than the control group mice, suggesting the impairment of spatial learning and memory (Figures 2(d) and 2(e)). A meta-analysis study suggested that cerebral perfusion reduction, higher cortisol levels, low levels of neurotrophic factors, and amygdala volume reduction may be potential biological markers in PSD patients [10]. Consistent with this report, corticosterone levels in serum were significantly increased (Figure 2(f)), and BDNF expression in the nucleus accumbens was markedly reduced (Supplementary Figure S1) in MCAO mice exposed to restraint stress as compared to those in the control mice. Therefore, the PSD animal model used in this study, a combination model with MCAO and spatial restraint stress, was a reliable chronic animal model of depression after stroke.

The molecular pathogenesis of PSD is predicted to involve multiple pathways, such as neuroinflammation, disturbed cellular plasticity, neuroendocrine dysregulation, and neurodegeneration [8, 11, 12]. Many studies have demonstrated that proinflammatory cytokines have an important role in the development of PSD [13, 14]. Inflammatory cytokines reduce serotonin levels via the upregulation of IDO1 [14], which induces depressive-like behaviors [15, 16]. In this study, we observed an increased IDO1 immunoreactivity in the nucleus accumbens, hippocampus, and hypothalamus, but not in the striatum of PSD mice (Figure 3). It has been reported that the nucleus accumbens is the center of reward and learning plays an important role in the pathophysiology of depression [23]. The hippocampus has been suggested to be involved in the pathophysiology of cognitive impairment in patients suffering from depressive disorders [24]. The hypothalamus is affected by stress and depression via the neuroendocrine system [25]. Basically, IDO1 is the initial enzyme that converts tryptophan to kynurenine which may lead to the production of neuroactive metabolites such as kynurenic acid (KA), 3-hydroxykynurenine (3-HK), and QUIN [26]. Recently, it has been reported that the kynurenine pathway becomes more active with age and the 3-HK level is positively associated with depression in nondemented women over 50 years of age [27].

While most brain cells such as neurons, astrocytes, and microglia can metabolize tryptophan to kynurenine, conversion of kynurenine to kynurenic acid occurs mainly in astrocytes, and the production of QUIN occurs mainly in activated microglia [28]. Under normal conditions, kynurenic acid produced in astrocytes is mostly involved in maintaining brain homeostasis [29]. However, during neuroinflammatory conditions, the kynurenine metabolism shifts toward increased production of QUIN in the microglia [30]. Therefore, it is possible that activated microglia with induced IDO1 producing the neurotoxic QUIN can affect PSD pathogenesis. We found that IDO1 expression occurred primarily in microglial cells in PSD mice (Figure 4(c)). Moreover, aripiprazole-treated PSD mice had markedly less IDO(+)/Iba-1(+) cells than untreated PSD mice (Figure 7), and the HAAO and QUIN levels were reduced by the aripiprazole treatment (Figure 8). Therefore, an increase in microglial IDO1 may cause the increased levels of HAAO and QUIN, which have been hypothesized as important factors in PSD progression.

However, IDO1 is not the only rate-limiting enzyme in the kynurenine pathway. The predominantly hepatic enzyme tryptophan-2,3-dioxygenase (TDO) is responsible for the initial step of the kynurenine pathway, metabolizing tryptophan to N-formylkynurenine, which is subsequently metabolized to kynurenine under normal homeostatic conditions. But under proinflammatory conditions or after experimental administration of LPS, the extrahepatic enzyme IDO is expressed in both the periphery and the brain, where it increases the production of kynurenine [31]. Because PSD is related to neuroinflammatory disorders, we measured IDO instead of TDO. However, TDO expression claimed to be restricted to the liver has also been found in other organs including the brain [32]. Hence, much still remains to be investigated to understand the respective roles of TDO and IDO in the brain.

We also observed higher levels of HAAO and QUIN in PSD mouse brains as compared to those in control mouse brains (Figure 5). Because it is astrocytes that mostly produce kynurenic acid from kynurenine whereas microglia convert kynurenine into 3-HK and QUIN [29, 30], the increase in microglial IDO1 expression is expected to increase the levels of 3-HK and QUIN but not kynurenic acid.

It was reported that the neurotoxic kynurenine metabolism was increased in the hippocampus and associated with distinct depressive behaviors during inflammation [33] and kynurenine 3-monooxygenase (KMO) was implicated in antidepressant-responsive depressive-like behaviors and monoaminergic dysfunctions [34]. Among the neurotoxic kynurenine metabolites, QUIN, an *N*-methyl-D-aspartate (NMDA) receptor agonist, can precipitate oxidative damage and elevate the potential for glutamate excitotoxicity that can cause neuronal damage and associated behavioral changes [21, 22]. Therefore, it is possible that neuronal cell death caused by an increased neurotoxic QUIN production may lead to depressive-like behavior and cognitive impairment in this PSD model. The ability of elevated QUIN concentrations to cause an overactivation of NMDA receptors may contribute to the hippocampal atrophy and hypothalamic-pituitary adrenal axis overactivity commonly reported in individuals with major depression [35].

The KMO-catalyzed reaction in microglia is the rate-limiting step in the kynurenine pathway, and its product, 3-HK, is believed to be neurotoxic due to increased ROS generation in neuronal apoptosis [36, 37]. QUIN neurotoxicity may be attributed to the generation of ROS [21, 22]. Lipid peroxidation produced by QUIN could also be attenuated by antioxidants, demonstrating that both free radical formation and NMDA receptor activation contributed to QUIN-induced oxidative damage [38]. So far, it has been known that oxidative stress is implicated in the pathology of depression in humans and in respective animal models [39, 40].

In this study, ROS production also significantly increased in the nucleus accumbens, hippocampus, and hypothalamus of PSD mice (Figure 9). We observed that aripiprazole treatment led to the attenuation of oxidative stress in PSD (Figure 9). Therefore, oxidative stress plays an important role in the pathogenesis of depressive-like symptoms and cognitive impairment following stroke. Interestingly, it has been reported that aripiprazole inhibits ROS generation, which is a remarkable antioxidant activity with a potential application in schizophrenia [41]. Aripiprazole is a third-generation atypical antipsychotic drug that is a partial agonist of the dopaminergic D_2_ receptor and the serotonin 5-HT_1A_ and 5-HT_7_ receptors [42]. Aripiprazole acts as a dopamine-serotonin system stabilizer that is used as an adjunct therapy for major depressive disorders [43, 44], typically applied in combination with selective serotonin reuptake inhibitors (SSRI). The combination therapy consisting of SSRI and low doses of aripiprazole is also an effective treatment regimen for patients with poststroke emotional disorders and impaired cognitive function [43, 45]. In our previous work, aripiprazole treatment resulted in improvement of all depressive and cognitive impairment behaviors via neuroprotection and neurogenesis in mice following an ischemic stroke and unpredictable chronic mild stress [6, 46] and exerted a neuroprotective effect in dopaminergic neuronal cells, potentially improving behavioral function following ischemic stroke [47]. Consistent with the earlier reports, aripiprazole ameliorated depressive-like behaviors and the impairment of cognitive function in the combination model with MCAO and spatial restraint stress (Figure 6).

## 5. Conclusion

The results of this study indicated the importance of IDO1 and IDO1-dependent neurotoxic kynurenine metabolites in microglia as a pathogenic mechanism of PSD in a mouse model induced by a combination of MCAO and spatial restraint stress. Moreover, the beneficial effect of the antipsychotic drug aripiprazole on depressive-like behavior and cognitive impairment caused by PSD may be mediated by inhibiting the kynurenine metabolism. Although further studies are needed to better understand the underlying mechanisms, our findings improve the knowledge about IDO1 and IDO1-dependent neurotoxic kynurenine metabolites as possible regulators leading to the development of PSD. Future studies should be conducted not only on the manipulation of IDO1, QUIN, and ROS for therapeutic purposes but also on the use of IDO1-dependent neurotoxic kynurenine metabolites as PSD biomarkers for early detection in evidence-based PSD management.

## Figures and Tables

**Table 1:** IDO1 immunoreactivity in the brain of PSD group mice.

	IDO^−^/marker^+^ cells (%)			IDO^+^/marker^+^ cells (%)		
	IDO^−^/NeuN^+^	IDO^−^/GFAP^+^	IDO^−^/Iba-1^+^	IDO^+^/NeuN^+^	IDO^+^/GFAP^+^	IDO^+^/Iba-1^+^
NAc	68.15 ± 5.09	74.39 ± 4.08	36.16 ± 5.43	31.85 ± 5.09	25.61 ± 4.08	63.84 ± 5.43
Hippo	77.87 ± 5.20	61.77 ± 3.52	35.10 ± 4.06	22.13 ± 5.20	38.23 ± 3.52	64.90 ± 4.06
Hypo	67.20 ± 2.87	54.55 ± 3.21	33.79 ± 2.45	32.80 ± 2.87	45.45 ± 3.21	66.21 ± 2.45

## Data Availability

The data used to support the findings of this study are available from the corresponding author upon request.

## Supplementary Materials

Supplementary Materials Supplementary Figure S1: effect of aripiprazole on the BDNF expression in the brain of PSD mice. (A) Representative photomicrographs of immunofluorescent staining of BDNF in the nucleus accumbens (NAc) of control (Con), sham (PSD), and aripiprazole-treated mice (PSD + APZ). BDNF was labeled with green fluorescence, and nuclear DNA was labeled with blue fluorescence by DAPI. Scale bar = 100 *μ*m. (B) Quantification graphs of BDNF fluorescence intensity. Data are expressed as mean ± SEM (*N* = 4). ^∗^ *P* < 0.05 vs. control; ^#^ *P* < 0.05 vs. PSD.
