# Supplementary material for: Indoleamine 2,3-Dioxygenase-Dependent Neurotoxic Kynurenine Metabolism Contributes to Poststroke Depression Induced in Mice by Ischemic Stroke along with Spatial Restraint Stress
Source: Oxid Med Cell Longev. 2018 Dec 30;2018:2413841. doi: 10.1155/2018/2413841 (PMC6332926; doi:10.1155/2018/2413841)

Supplementary Figure S1. Effect of aripiprazole on the BDNF expression in the brain of PSD mice. (A) Representative photomicrographs of immunofluorescent staining of BDNF in the nucleus accumbens (NAc) of control (Con), sham (PSD), and aripiprazole-treated mice (PSD+APZ). BDNF was labeled with green fluorescence and nuclear DNA was labeled with blue fluorescence by DAPI. Scale bar = 100 μm. **(B)** Quantification graphs of BDNF fluorescence intensity. Data are expressed as the mean ± SEM (N=4). * $P < 0.05$ vs. control; # $P < 0.05$ vs. PSD.

Supplementary Figure S1

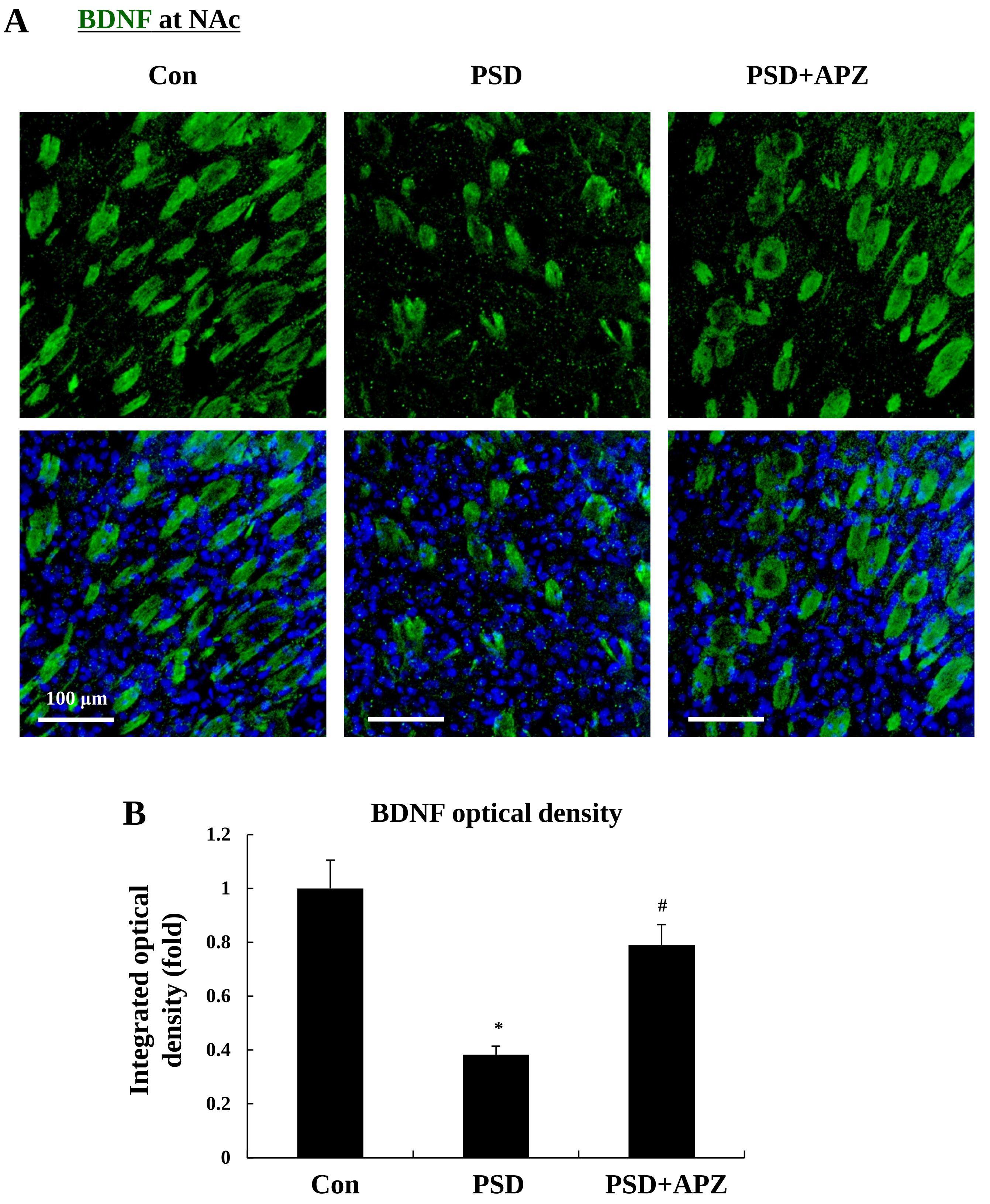

Supplement: Supplementary Materials — Supplementary Figure S1: effect of aripiprazole on the BDNF expression in the brain of PSD mice. (A) Representative photomicrographs of immunofluorescent staining of BDNF in the nucleus accumbens (NAc) of control (Con), sham (PSD), and aripiprazole-treated mice (PSD + APZ). BDNF was labeled with green fluorescence, and nuclear DNA was labeled with blue fluorescence by DAPI. Scale bar = 100 μm. (B) Quantification graphs of BDNF fluorescence intensity. Data are expressed as mean ± SEM (N = 4). ∗ P < 0.05 vs. control; # P < 0.05 vs. PSD. [file 2413841.f1.pdf]
